# Prevalence of Chronic Diseases, Depression, and Stress Among US Childcare Professionals During the COVID-19 Pandemic

**DOI:** 10.5888/pcd19.220132

**Published:** 2022-09-22

**Authors:** Jad A. Elharake, Mehr Shafiq, Ayse Cobanoglu, Amyn A. Malik, Madeline Klotz, John Eric Humphries, Thomas Murray, Kavin M. Patel, David Wilkinson, Inci Yildirim, Rachel Diaz, Rosalia Rojas, Anael Kuperwajs Cohen, Aiden Lee, Saad B. Omer, Walter S. Gilliam

**Affiliations:** 1 Yale School of Medicine, New Haven, Connecticut; 2 Yale Institute for Global Health, New Haven, Connecticut; 3 Mailman School of Public Health, Columbia University, New York, New York; 4 Yale Child Study Center, Yale School of Medicine, New Haven, Connecticut; 5 Human Development and Family Studies, Michigan State University, East Lansing, Michigan; 6 Department of Economics, Yale University, New Haven, Connecticut; 7 Department of Pediatrics, Yale School of Medicine, New Haven, Connecticut; 8 Tobin Center for Economic Policy, Yale University, New Haven, Connecticut; 9 Department of Epidemiology of Microbial Diseases, Yale School of Public Health, New Haven, Connecticut; 10 Yale School of Nursing, New Haven, Connecticut

## Abstract

**Introduction:**

Given their central role in supporting children’s development, childcare professionals’ overall physical and mental health is important. We evaluated the prevalence of chronic diseases, depression, and stress levels during the COVID-19 pandemic among US childcare professionals.

**Methods:**

Data were obtained from US childcare professionals (N = 81,682) through an online survey from May 22, 2020, through June 8, 2020. We used multivariable logistic and linear regression models to assess the association of sociodemographic characteristics with 4 physical health conditions (asthma, heart disease, diabetes, and obesity), depression, and stress weighted to national representativeness.

**Results:**

For physical health conditions, 14.3% (n = 11,717) reported moderate to severe asthma, 6.5% (n = 5,317) diabetes, 4.9% (n = 3,971) heart disease, and 19.8% (n = 16,207) obesity. For mental health, 45.7% (n = 37,376) screened positive for depression and 66.5% (n = 54,381) reported moderate to high stress levels. Race, ethnicity, and sex/gender disparities were found for physical health conditions but not mental health of childcare professionals during the COVID-19 pandemic.

**Conclusion:**

Our findings highlighted that childcare professionals’ depression rates during the pandemic were higher than before the pandemic, and depression, stress, and asthma rates were higher than rates among US adults overall during the pandemic. Given the essential work childcare professionals provided during the pandemic, policy makers and public health officials should consider what can be done to support their physical and mental health.

**Summary**

**What is already known on this topic?**

No national research has been published on the physical or mental health of childcare professionals during the COVID-19 pandemic.

**What is added by this report?**

Childcare professionals had a higher rate of depression during the pandemic than before the pandemic; rates of asthma, depression, and stress were greater among childcare professionals than among US adults overall during the pandemic. We found disparities in chronic disease rates by race, ethnicity, and sex/gender.

**What are the implications for public health practice?**

Efforts should be directed toward developing effective and scalable interventions for improving the physical and mental health of childcare professionals and addressing stressors that may undermine their well-being.

**MEDSCAPE CME**

In support of improving patient care, this activity has been planned and implemented by Medscape, LLC and *Preventing Chronic Disease*. Medscape, LLC is jointly accredited by the Accreditation Council for Continuing Medical Education (ACCME), the Accreditation Council for Pharmacy Education (ACPE), and the American Nurses Credentialing Center (ANCC), to provide continuing education for the healthcare team.

Medscape, LLC designates this Journal-based CME activity for a maximum of 1.00 AMA PRA Category 1 Credit(s)™. Physicians should claim only the credit commensurate with the extent of their participation in the activity.

Successful completion of this CME activity, which includes participation in the evaluation component, enables the participant to earn up to 1.0 MOC points in the American Board of Internal Medicine’s (ABIM) Maintenance of Certification (MOC) program. Participants will earn MOC points equivalent to the amount of CME credits claimed for the activity. It is the CME activity provider’s responsibility to submit participant completion information to ACCME for the purpose of granting ABIM MOC credit.

Release date: September 22, 2022; Expiration date: September 22, 2023

**Learning Objectives**

Upon completion of this activity, participants will be able to:

1. Assess common chronic illnesses among US childcare professionals
2. Distinguish racial/ethnic groups at higher risk for chronic illness among US childcare professionals
3. Distinguish the rate of positive screening for depression among US childcare professionals
4. Evaluate variables associated with higher stress among US childcare professionals

**EDITOR**

Ellen Taratus, MS

Senior Editor

Preventing Chronic Disease

Atlanta, GA

**CME AUTHOR**

Charles P. Vega, MD

Health Sciences Clinical Professor of Family Medicine

University of California, Irvine School of Medicine

Charles P. Vega, MD, has the following relevant financial relationships:

Consultant or advisor for: GlaxoSmithKline; Johnson & Johnson Pharmaceutical Research & Development, LLC

**AUTHORS**

Jad A. Elharake, MPH

Yale School of Medicine, New Haven, CT; Yale Institute for Global Health, New Haven, CT

Mehr Shafiq, MPH

Yale Institute for Global Health, New Haven, CT; Mailman School of Public Health, Columbia University, New York, NY

Ayse Cobanoglu, PhD

Yale Child Study Center, Yale School of Medicine, New Haven, CT

Amyn A. Malik, PhD, MBBS, MPH

Yale School of Medicine, New Haven, CT; Yale Institute for Global Health, New Haven, CT

Madeline Klotz, BA

Human Development and Family Studies, Michigan State University, East Lansing, MI

John Eric Humphries, PhD

Department of Economics, Yale University, New Haven, CT

Thomas Murray, MD, PhD

Department of Pediatrics, Yale School of Medicine, New Haven, CT

Kavin M. Patel, MD

Yale School of Medicine, New Haven, CT

David Wilkinson, JD

Tobin Center for Economic Policy, Yale University, New Haven, CT

Inci Yildirim, MD, PhD, MSc

Yale Institute for Global Health, New Haven, CT; Department of Pediatrics, Yale School of Medicine, New Haven, CT

Rachel Diaz, BA

Tobin Center for Economic Policy, Yale University, New Haven, CT

Rosalia Rojas, BA

Yale Child Study Center, Yale School of Medicine, New Haven, CT

Anael Kuperwajs Cohen, BA

Yale Child Study Center, Yale School of Medicine, New Haven, CT

Aiden Lee

Department of Economics, Yale University, New Haven, CT

Saad B. Omer, MBBS, MPH, PhD

Yale School of Medicine, New Haven, CT; Yale Institute for Global Health, New Haven, CT; Yale School of Public Health, New Haven, CT; Yale School of Nursing, New Haven, CT

Walter S. Gilliam, PhD

Yale Child Study Center, Yale School of Medicine, New Haven, CT

## Introduction

Approximately 1.1 million paid and registered childcare professionals in the US provide care for 10 million children in center-based and home-based settings (1). Childcare professionals (eg, childcare center workers, family childcare providers, nannies) make up a socially vulnerable workforce (2). As of 2019, this workforce consisted largely of women (94%) and in many childcare sectors, members of racial and ethnic minority groups (40%), immigrants (22%), and low-income individuals (average salary of $16,443, 67% below the national average) (3,4). Childcare professionals also face challenging work conditions, including long hours and physical and emotional demands (5), leading to staff turnover, absenteeism, poor physical health conditions, high rates of burnout, emotional exhaustion, and mental health problems (6). Additionally, the unpredictability and impact of the COVID-19 pandemic has exacerbated the financial and work-related stress faced by childcare professionals (7).

No national research has been published on the physical or mental health (eg, depression, stress) of childcare professionals during the COVID-19 pandemic. Prepandemic studies on childcare professionals reported a diabetes rate of 7.3% (8), obesity rates ranging from 34% to 66.3% (8–11), and clinical depression rates ranging from 16.0% to 36.1% (11–15). The overall rates among US adult women were 8.7% for diabetes (16), 41.9% for obesity (17), and 9.6% for depression (18) before the pandemic, and 27.8% to 32.8% for depression during the pandemic (19–21). Only 1 study focused on the stress levels of childcare professionals during the pandemic; conducted in Indiana, it found that 63% of childcare professionals had moderate to high stress levels (7), almost twice the national estimate of 37% among US adults during the pandemic (22). Before the pandemic, 36.8% to 62.1% of childcare professionals were reported to be experiencing moderate to high levels of stress (7,10,23).

Given that the mental and physical well-being of childcare professionals is associated with children’s academic and emotional learning outcomes (5,15,23), it is imperative to understand and address the condition of childcare professionals during the pandemic and beyond. Although relatively small-scale studies documented the mental health status of early childhood educators before the COVID-19 pandemic (5,8–11,14,15,23), little is known about their physical and mental health status during the pandemic. This study evaluated the prevalence of chronic diseases, depression, and stress levels during the COVID-19 pandemic among US childcare professionals.

## Methods

Data were collected from self-identifying childcare professionals through an online Qualtrics survey distributed from May 22, 2020, through June 8, 2020, approximately 10 to 13 weeks into the COVID-19 pandemic, through various contact lists of individuals associated with the childcare industry, as described previously (24). Inclusion criteria were participants who self-identified as childcare professionals working in childcare before or during the pandemic; consented to the study; were aged 18 years or older; and resided in a US state or the District of Columbia. Of the 94,390 individuals who accessed the survey, 82,613 satisfied inclusion criteria and 81,682 (98.9%) provided data necessary for analyses. Participants were offered entry into a raffle for 1 of 20 gift cards valued at $500 each. The research protocol was approved by the Yale University Institutional Review Board as a Category 2(ii) exempt protocol (#2000028232).

### Variables

**Chronic diseases and health conditions.** The survey asked about 10 chronic diseases and physical health conditions, identified as risk factors for COVID-19 complications by the Centers for Disease Control and Prevention at the time of the survey (25). Respondents indicated which applied to them: chronic lung disease/chronic obstructive pulmonary disease (COPD), chronic/severe kidney disease, liver disease, heart disease, immune-compromising conditions (such as immune deficiencies or bone marrow/organ transplant), immunosuppressive treatments of cancer/inflammatory disease (such as lupus or rheumatoid arthritis), smoking, diabetes, asthma (moderate to severe), and obesity. The highest prevalence in our sample was for asthma, diabetes, heart disease, and obesity; therefore, we selected these conditions for further analysis. We analyzed the prevalence of the remaining 6 physical health conditions by sociodemographic characteristics.

**Depressive symptoms.** The 10-item Center for Epidemiological Studies–Depression (CES-D-10) scale is a reliable and valid self-report scale designed to measure depressive symptomatology and screen for major depression (26). Items assess depression-related symptoms experienced in the previous week (0 = rarely or none of the time; 1 = some or little of the time; 2 = occasionally or a moderate amount of the time; 3 = all of the time), such as restless sleep, poor appetite, and feelings of loneliness. We reverse coded positively stated items (eg, “I felt hopeful about the future”) before calculating summary scores (possible range, 0–30). We calculated summary scores by totaling all items scored. As in other studies (20), summary scores greater than or equal to 10 were considered positive for depression.

**Stress.** The 10-item Perceived Stress Scale (PSS-10) is a validated short-form version of the PSS, the most widely used psychological instrument for measuring the perception of stress (27). Questions ask about feelings and thoughts during the previous month and are rated on a 5-point Likert scale (0 = never; 1 = almost never; 2 = sometimes; 3 = fairly often; 4 = very often) (27). Positively stated items (eg, “felt that things were going your way”) were reverse coded before calculating summary scores (range, 0–40). We calculated summary scores by totaling all items scored. Summary scores ranging from 0 to 13 are considered low stress; 14 to 26, moderate stress; and 27 to 40, high stress (27).

**Sociodemographic factors. **Respondents were asked to provide information on their race and ethnicity, with items worded identically to the most recent US Census questionnaire. Options for race were White, Black/African American, American Indian/Alaska Native, Native Hawaiian/Pacific Islander, and Asian; respondents who selected more than 1 race were coded as multiracial. Additionally, ethnicity (Hispanic, Latino, or Spanish origin versus not), access to medical insurance (yes or no), sex/gender (female, male, nonbinary, prefer to self-describe as something else, prefer not to answer), age, and childcare program type (for-profit center, nonprofit agency center, school-based center, Head Start or Early Head Start, other center-based, drop-in childcare, home-based or family childcare, nanny or in-home childcare) were considered in the analysis.

### Data weighting and missing data analysis and treatment

The sample was weighted to national representativeness for US childcare professionals by state, age, race, and ethnicity based on the 2019 American Community Survey (28), with the top and bottom 5% of the weights trimmed to reduce sampling variance.

Analysis of missing data included visual examination of missing data patterns and descriptive measures of missing values. Chronic diseases and health conditions had 9.7% missing values, while CES-D-10 and PSS-10 scores had 36.1% and 37.3% missing values, respectively. Missingness in covariates ranged from 0.5% to 16%. We used Little’s missing completely at random test to examine the missing data mechanism, and results suggested that the data were not missing completely at random (χ^2^_9_ = 1,515.8; *P* < .001) (29). We used multiple imputation to address missingness (30). Variables used for imputation included race, ethnicity, sex/gender, age, childcare program type, access to medical insurance, and all outcome variables (CES-D-10, PSS-10, diabetes, heart disease, asthma, and obesity). Weight was also incorporated to fit imputation models. To ensure the precision and replicability of point estimates, we imputed 20 data sets using the fully conditional specification imputation method (31) and reported the pooled results from the 20 data sets.

### Statistical analysis

We used descriptive statistics to present all variables. We used separate multivariable logistic regression models to assess the association of covariates (age, race, ethnicity, sex/gender, program type, and medical insurance) with the prevalence of heart disease, asthma, diabetes, and obesity. We used separate multivariable linear regression models to assess the association of these covariates with self-reported depression and stress scores. Significance was set to α = .05 (2-tailed). Effect size of odds ratios (ORs) were interpreted as being very small (<1.44), small (1.44–2.47), medium (2.48–4.26), or large (≥4.27) (32). We conducted all analyses in SPSS Statistics version 28.0.1 (IBM Corp) and R version 4.1.1 (R Foundation for Statistical Computing).

## Results

Of the total sample (N = 81,682), the mean age was 42.1 years (SD, 14.1 y). Across racial categories, 63.8% (n = 52,164) were White, 14.5% (n = 11,837) Black/African American, 3.6% (n = 2,949) Asian, 3.6% (n = 2,944) multiracial, 1.9% (n = 1,582) American Indian/Alaska Native, 0.6% (n = 491) Native Hawaiian/Pacific Islander, and 11.9% (n = 9,731) preferred not to identify their race; 21.7% (n = 17,753) identified as Hispanic. Also, 96.4% (n = 78,725) of the sample identified as female, 2.5% (n = 2,033) as male, and 0.3% (n = 225) as nonbinary. Most (76.5%) respondents worked in childcare centers; of these, 24.5% (n = 19,976) worked in for-profit centers. Of the total sample, 89.2% (n = 72,890) reported access to medical insurance (Table 1).

**Table 1:** Prevalence of Physical Health Conditions Among US Childcare Professionals, by Sociodemographic Characteristics, 2020^a^

Characteristic	Total	Physical health condition			
		Asthma	Heart disease	Diabetes	Obesity
**Total^b^**	81,682	11,717 (14.3)	3,971 (4.9)	5,317 (6.5)	16,207 (19.8)
**Age, mean (SD), y**	42.1 (14.1)	40.9 (14.1)	50.3 (13.8)	51.3 (12.8)	44.0 (13.3)
**Race**					
American Indian/Alaska Native	1,582 (1.9)	298 (18.8)	91 (5.8)	178 (11.2)	330 (20.9)
Asian	2,949 (3.6)	325 (11.0)	121 (4.1)	195 (6.6)	195 (6.6)
Black/African American	11,837 (14.5)	1,787 (15.1)	674 (5.7)	1,209 (10.2)	2,587 (21.9)
Multiracial	2,944 (3.6)	638 (21.7)	179 (6.1)	130 (4.4)	703 (23.9)
Native Hawaiian/Pacific Islander	491 (0.6)	67 (13.6)	21 (4.3)	53 (10.9)	77 (15.7)
Prefer to not answer	9,731 (11.9)	1,189 (12.2)	364 (3.7)	606 (6.2)	1,410 (14.5)
White	52,164 (63.8)	7,413 (14.2)	2,520 (4.8)	2,946 (5.6)	10,905 (20.9)
**Hispanic ethnicity**					
No	61,806 (75.7)	9,099 (14.7)	3,207 (5.2)	4,043 (6.5)	13,121 (21.2)
Yes	17,753 (21.7)	2,362 (13.3)	676 (3.8)	1,112 (6.3)	2,723 (15.3)
Prefer to not answer	2,139 (2.6)	255 (11.9)	88 (4.1)	163 (7.6)	363 (17.0)
**Sex/gender**					
Female	78,725 (96.4)	11,358 (14.4)	3,789 (4.8)	5,106 (6.5)	15,780 (20.0)
Male	2,033 (2.5)	231 (11.4)	139 (6.8)	149 (7.3)	267 (13.1)
Nonbinary	225 (0.3)	51 (22.8)	14 (6.3)	11 (4.8)	51 (22.7)
Prefer to not answer	715 (0.9)	76 (10.6)	29 (4.1)	52 (7.3)	109 (15.2)
**Medical insurance**					
No	8,808 (10.8)	1,012 (11.5)	327 (3.7)	424 (4.8)	1,551 (17.6)
Yes	72,890 (89.2)	10,705 (14.7)	3,644 (5.0)	4,893 (6.7)	14,656 (20.1)
**Program type **					
Center-based					
For-profit center	19,976 (24.5)	2,861 (14.3)	1,050 (5.3)	1,222 (6.1)	4,133 (20.7)
School-based	10,604 (13.0)	1,529 (14.4)	450 (4.2)	465 (4.4)	1,729 (16.3)
Head Start/Early Head Start	8,506 (10.4)	1,309 (15.4)	370 (4.4)	637 (7.5)	1,992 (23.4)
Drop-in center	1,596 (2.0)	220 (13.8)	49 (3.1)	69 (4.3)	210 (13.1)
Nonprofit agency center	15,875 (19.4)	2,336 (14.7)	802 (5.1)	1,076 (6.8)	3,588 (22.6)
Other center-based	5,949 (7.3)	945 (15.9)	267 (4.5)	352 (5.9)	1,073 (18.0)
Home-based/family childcare	18,078 (22.1)	2,348 (13.0)	946 (5.2)	1,435 (7.9)	3,309 (18.3)
Nanny/home visiting	1,115 (1.4)	169 (15.2)	37 (3.3)	61 (5.4)	174 (15.6)

a Data were collected from self-identifying childcare professionals through an online Qualtrics survey distributed from May 22, 2020, through June 8, 2020. All values are number (percentage) unless otherwise indicated.

b Because of sample weighting, frequencies may not add exactly to totals.

### Chronic diseases and physical health conditions

Of the 10 chronic diseases and physical health conditions considered, we found the highest rates for asthma (14.3%; n = 11,717), diabetes (6.5%; n = 5,317), heart disease (4.9%; n = 3,971), and obesity (19.8%; n = 16,207) (Table 1).

#### Asthma

Compared with White respondents, American Indian/Alaska Native (OR, 1.44; 95% CI, 1.23–1.69), Black/African American (OR, 1.09; 95% CI, 1.02–1.16), and multiracial (OR, 1.62; 95% CI, 1.47–1.79) respondents had higher odds of having asthma, while controlling for other covariates, whereas Asian (OR, 0.74; 95% CI, 0.64–0.84) respondents had lower odds of having asthma (Table 2). Respondents who identified as Hispanic had lower odds (OR, 0.90; 95% CI, 0.85–0.96) of having asthma than those who did not identify as Hispanic, while controlling for other covariates. Compared with female respondents, respondents who identified as nonbinary had higher odds of asthma (OR, 1.58; 95% CI, 1.11–2.25). Respondents without medical insurance had lower odds of having asthma (OR, 0.74; 95% CI, 0.68–0.80) than respondents with medical insurance, while controlling for other covariates.

**Table 2:** Logistic Regression of Physical Health Conditions and Sociodemographic Characteristics Among US Childcare Professionals, 2020^a^

Characteristic	Asthma	Heart disease	Diabetes	Obesity
**Age**	0.99 (0.99–0.99)^b^	1.04 (1.04–1.05)^b^	1.05 (1.05–1.06)^b^	1.01 (1.01–1.01)^b^
**Race**				
American Indian/Alaska Native	1.44 (1.23–1.69)^b^	1.42 (1.11–1.82)^b^	2.34 (1.96–2.80)^b^	1.11 (0.95–1.29)
Asian	0.74 (0.64–0.84)^b^	0.89 (0.72–1.11)	1.34 (1.11–1.62)^b^	0.27 (0.22–0.31)^b^
Black/African American	1.09 (1.02–1.16)^b^	1.14 (1.03–1.26)^b^	1.86 (1.71–2.03)^b^	1.01 (0.96–1.07)
Multiracial	1.62 (1.47–1.79)^b^	1.74 (1.46–2.08)^b^	1.07 (0.87–1.31)	1.30 (1.18–1.43)^b^
Native Hawaiian/Pacific Islander	1.00 (0.73–1.37)	0.88 (0.53–1.45)	1.88 (1.34–2.64)^b^	0.76 (0.58–0.99)^b^
Prefer not to answer	0.91 (0.83–0.99)	0.95 (0.81–1.11)	1.09 (0.96–1.23)	0.78 (0.72–0.85)^b^
White	1 [Reference]	1 [Reference]	1 [Reference]	1 [Reference]
**Hispanic**				
No	1 [Reference]	1 [Reference]	1 [Reference]	1 [Reference]
Yes	0.90 (0.85–0.96)^b^	0.95 (0.85–1.06)	1.34 (1.22–1.47)^b^	0.75 (0.71–0.80)^b^
Prefer to not answer	0.90 (0.76–1.07)	0.82 (0.61–1.12)	1.23 (0.98–1.54)	0.93 (0.80–1.08)
**Sex/gender**				
Female	1 [Reference]	1 [Reference]	1 [Reference]	1 [Reference]
Male	0.74 (0.63–0.88)^b^	1.49 (1.22–1.80)^b^	1.18 (0.96–1.44)	0.62 (0.53–0.73)^b^
Nonbinary	1.58 (1.11–2.25)^b^	1.97 (1.01–3.84)^b^	1.06 (0.45–2.48)	1.32 (0.90–1.93)
Prefer to not answer	0.80 (0.55–1.17)	0.93 (0.53–1.65)	0.99 (0.66–1.50)	0.82 (0.63–1.07)
**Medical insurance**				
Yes	1 [Reference]	1 [Reference]	1 [Reference]	1 [Reference]
No	0.74 (0.68–0.80)^b^	0.89 (0.77–1.02)	0.81 (0.72–0.92)^b^	0.94 (0.88–1.00)^b^
**Program type**				
For-profit center	1 [Reference]	1 [Reference]	1 [Reference]	1 [Reference]
Home-based/family childcare	0.94 (0.88–1.01)	0.83 (0.74–0.92)^b^	0.99 (0.89–1.10)	0.85 (0.80–0.91)^b^
Nanny/home visiting	1.02 (0.84–1.25)	0.89 (0.58–1.36)	1.27 (0.91–1.77)	0.82 (0.67–1.00)^b^
Nonprofit agency center	1.05 (0.98–1.12)	0.87 (0.78–0.97)^b^	1.01 (0.91–1.11)	1.10 (1.03–1.17)^b^
School-based	0.99 (0.91–1.08)	0.90 (0.78–1.04)	0.78 (0.69–0.88)^b^	0.81 (0.75–0.87)^b^
Head Start/Early Head Start	1.08 (0.99–1.17)	0.89 (0.77–1.02)	1.21 (1.08–1.35)^b^	1.28 (1.19–1.37)^b^
Drop-in center	0.92 (0.78–1.09)	0.86 (0.59–1.24)	0.95 (0.69–1.30)	0.69 (0.57–0.84)^b^
Other center-based	1.14 (1.03–1.26)^b^	0.91 (0.77–1.06)	1.00 (0.87–1.16)	0.89 (0.81–0.98)^b^

a Data were collected from self-identifying childcare professionals through an online Qualtrics survey distributed from May 22, 2020, through June 8, 2020. All values are odds ratio (95% CI).

b *P* < .05; determined by logistic regression.

#### Diabetes

Compared with White respondents, American Indian/Alaska Native (OR, 2.34; 95% CI, 1.96–2.80), Asian (OR, 1.34; 95% CI, 1.11–1.62), Black/African American (OR, 1.86; 95% CI, 1.71–2.03), and Native Hawaiian/Pacific Islander (OR, 1.88; 95% CI, 1.34–2.64) respondents had higher odds of having diabetes, while controlling for other covariates (Table 2). Respondents who identified as Hispanic had higher odds (OR, 1.34; 95% CI, 1.22–1.47) of having diabetes than those who did not identify as Hispanic, while controlling for other covariates. Respondents without medical insurance had lower odds of having diabetes (OR, 0.81; 95% CI, 0.72–0.92) than those with medical insurance, while controlling for other covariates.

#### Heart disease

Compared with White respondents, American Indian/Alaska Native (OR, 1.42; 95% CI, 1.11–1.82), Black/African American (OR, 1.14; 95% CI, 1.03–1.26), and multiracial (OR, 1.74; 95% CI, 1.46–2.08) respondents had higher odds of having heart disease, while controlling for ethnicity, sex/gender, age, medical insurance, and program type (Table 2). Male respondents (OR, 1.49; 95% CI, 1.22–1.80) and nonbinary respondents (OR, 1.97; 95% CI, 1.01–3.84) had higher odds of having heart disease than did female respondents.

#### Obesity

Compared with White respondents, multiracial (OR, 1.30; 95% CI, 1.18–1.43) respondents had higher odds of having obesity, while controlling for other covariates, whereas Asian (OR, 0.27; 95% CI, 0.22–0.31) and Native Hawaiian/Pacific Islander (OR, 0.76; 95% CI, 0.58–0.99) respondents had lower odds of having obesity (Table 2). Participants who identified as Hispanic had lower odds (OR, 0.75; 95% CI, 0.71–0.80) of having obesity than respondents who did not identify as Hispanic, while controlling for other covariates. Respondents without medical insurance had lower odds of having obesity (OR, 0.94; 95% CI, 0.88–1.00) than respondents with medical insurance, while controlling for other covariates.

#### Other physical health conditions

The prevalence of the remaining 6 chronic diseases and physical health conditions were 4.4% (n = 3,619) for smoking, 4.7% (n = 3,851) for immunosuppressive treatments of cancer/inflammatory disease, 2.3% (n = 1,884) for immune-compromising conditions, 1.0% (n = 814) for chronic lung disease/COPD, 0.7% (n = 562) for chronic/severe kidney disease, and 0.7% (n = 545) for liver disease (Supplemental Table 1 in Appendix). Overall, 26.2% (n = 21,398) of respondents reported at least 1 medically compromising condition, 9.7% (n = 7,962) reported 2 conditions, and 4.0% (n = 3,239) reported 3 or more conditions (Supplemental Table 2 in Appendix).

### Mental health

#### Depressive symptoms

Of the total sample, 45.7% (n = 37,376) of respondents screened positive for depression, with a mean score of 10.2 (SD, 6.0) (Table 3). For every 1-year increase in age, on average, the CES-D-10 summary score decreased (β = −0.11; 95% CI, −0.16 to −0.05) (Table 4). Compared with respondents who worked in for-profit centers, participants in home-based programs reported lower CES-D-10 summary scores (β = −2.30; 95% CI, −3.89 to −0.72) while controlling for other covariates (Table 4).

**Table 3:** Prevalence of Mental Health Outcomes, by Sociodemographic Characteristics, Among US Childcare Professionals, 2020^a^

Characteristic	Depression (CES-D-10)^b^			Stress (PSS-10)^c^			
	Mean (SD) score	Screened negative, no. (%)	Screened positive, no. (%)	Mean (SD) score	Low stress, no. (%)	Moderate stress, no. (%)	High stress, no. (%)
**Total**	10.2 (6.0)	44,322 (54.3)	37,376 (45.7)	17.5 (7.4)	27,317 (33.4)	34,752 (42.5)	19,629 (24.0)
**Race**							
American Indian/Alaska Native	10.5 (5.9)	776 (49.1)	805 (50.9)	17.9 (7.2)	518 (32.7)	679 (42.9)	385 (24.3)
Asian	9.9 (5.8)	1,659 (56.3)	1,290 (43.7)	18.2 (6.9)	773 (26.2)	1,402 (47.5)	774 (26.2)
Black/African American	9.8 (5.8)	6,990 (59.1)	4,847 (40.9)	16.5 (7.6)	4,669 (39.4)	4,265 (36.0)	2,904 (24.5)
Multiracial	11.3 (5.9)	1,330 (45.2)	1,614 (54.8)	18.4 (7.1)	816 (27.7)	1,357 (46.1)	771 (26.2)
Native Hawaiian/Pacific Islander	8.9 (5.2)	308 (62.7)	183 (37.3)	16.5 (7.5)	223 (45.3)	164 (33.4)	104 (21.3)
Prefer to not answer	10.4 (5.7)	5,183 (53.3)	4,548 (46.7)	17.6 (7.3)	3,156 (32.4)	4,029 (41.4)	2,547 (26.2)
White	10.2 (6.0)	28,075 (53.8)	24,089 (46.2)	17.6 (7.3)	17,163 (32.9)	22,856 (43.8)	12,145 (23.3)
**Hispanic**							
No	10.2 (6.0)	33,644 (54.4)	28,162 (45.6)	17.5 (7.4)	20,647 (33.4)	26,538 (42.9)	14,621 (23.7)
Yes	10.3 (5.8)	9,494 (53.5)	8,259 (46.5)	17.5 (7.3)	5,932 (33.4)	7,390 (41.6)	4,431 (25.0)
Prefer to not answer	10.5 (5.7)	1,184 (55.3)	955 (44.7)	17.4 (7.3)	739 (34.5)	823 (38.5)	577 (27.0)
**Sex/gender**							
Female	10.2 (6.0)	42,714 (54.3)	36,011 (45.7)	17.5 (7.4)	26,346 (33.5)	33,459 (42.5)	18,921 (24.0)
Male	10.1 (6.0)	1,169 (57.5)	864 (42.5)	17.0 (7.5)	667 (32.8)	883 (43.5)	482 (23.7)
Nonbinary	13.7 (6.3)	75 (33.5)	150 (66.5)	19.7 (7.3)	65 (29.0)	91 (40.5)	69 (30.5)
Prefer to not answer	10.2 (5.3)	364 (51.0)	351 (49.0)	17.1 (6.6)	239 (33.4)	319 (44.6)	158 (22.0)
**Health insurance**							
No	10.6 (6.0)	4,516 (51.3)	4,292 (48.7)	18.0 (7.5)	2,908 (33.0)	3,593 (40.8)	2,306 (26.2)
Yes	10.2 (5.9)	39,807 (54.6)	33,083 (45.4)	17.4 (7.4)	24,409 (33.5)	31,159 (42.7)	17,322 (23.8)
**Program type**							
For-profit center	10.6 (6.0)	10,707 (53.6)	9,269 (46.4)	18.3 (7.4)	6,193 (31.0)	8,400 (42.0)	5,383 (27.0)
Home-based/family childcare	8.6 (5.9)	11,552 (63.9)	6,526 (36.1)	15.4 (7.6)	7,748 (42.9)	7,100 (39.3)	3,230 (17.8)
Nanny/home visiting	11.0 (6.0)	533 (47.7)	581 (52.3)	18.7 (7.3)	291 (26.2)	470 (42.1)	353 (31.7)
Nonprofit agency center	10.7 (5.9)	8,163 (51.5)	7,712 (48.5)	18.0 (7.2)	4,959 (31.2)	7,245 (45.6)	3671 (23.1)
School-based	10.9 (5.9)	5,135 (48.4)	5,469 (51.6)	18.2 (7.1)	3,146 (29.6)	4,564 (43.1)	2,894 (27.3)
Head Start/Early Head Start	10.6 (5.7)	4,308 (50.6)	4,198 (49.4)	17.6 (7.0)	2,644 (31.1)	3,980 (46.8)	1,881 (22.2)
Drop-in center	11.9 (5.8)	795 (49.6)	801 (50.4)	18.9 (6.9)	522 (32.9)	620 (38.7)	454 (28.4)
Other center-based	10.5 (5.7)	3,129 (52.5)	2,820 (47.5)	17.8 (6.9)	1,816 (30.5)	2,371 (39.9)	1,762 (29.6)

Abbreviations: CES-D-10, Epidemiological Studies–Depression-10; PSS-10, Perceived Stress Scale-10.

a Data were collected from self-identifying childcare professionals through an online Qualtrics survey distributed from May 22, 2020, through June 8, 2020.

b CES-D-10 items assess depression-related symptoms experienced in the previous week (0 = rarely or none of the time; 1 = some or little of the time; 2 = occasionally or a moderate amount of the time; 3 = all of the time), such as restless sleep, poor appetite, and feelings of loneliness (26). Possible scores range from 0 to 30. Scores ≥10 were considered positive for depression.

c PSS-10 questions ask about feelings and thoughts during the previous month and are rated on a 5-point Likert Scale (0 = never; 1 = almost never; 2 = sometimes; 3 = fairly often; 4 = very often) (27). Possible scores range from 0 to 40; scores of 0 to 13 are considered low stress, 14 to 26 moderate stress, and 27 to 40 high stress.

**Table 4:** Linear Regression of CES-D-10, PSS-10, and Sociodemographic Characteristics Among US Childcare Professionals, 2020^a^

Characteristic	Depressive Symptoms (CES-D-10)		Stress (PSS-10)	
	β (95% CI)	SE	β (95% CI)	SE
**Age**	−0.11 (−0.16 to −0.05)^b^	0.03	−0.07 (−0.11 to −0.03)^b^	0.02
**Race**				
American Indian/Alaska Native	0.28 (−1.60 to 2.15)	0.90	0.14 (−1.53 to 1.82)	0.81
Asian	0.53 (−2.52 to 3.57)	1.45	−0.39 (−2.34 to 1.55)	0.93
Black/African American	−0.83 (−2.17 to 0.51)	0.64	−0.26 (−1.18 to 0.66)	0.44
Multiracial	0.11 (−1.91 to 2.13)	0.97	0.58 (−1.29 to 2.46)	0.90
Native Hawaiian/Pacific Islander	−0.60 (−5.21 to 4.01)	2.21	−1.09 (−4.59 to 2.41)	1.68
Prefer to not answer	0.16 (−1.83 to 2.16)	0.95	0.28 (−1.07 to 1.63)	0.65
White	1 [Reference]		1 [Reference]	
**Hispanic**				
No	1 [Reference]		1 [Reference]	
Yes	−0.46 (−1.67 to 0.75)	0.58	−0.33 (−1.50 to 0.84)	0.56
Prefer to not answer	−0.16 (−2.46 to 2.13)	1.10	0.08 (−2.62 to 2.79)	1.29
**Sex/gender**				
Female	1 [Reference]		1 [Reference]	
Male	−0.95 (−3.52 to 1.61)	1.23	−0.45 (−2.29 to 1.38)	0.88
Nonbinary	0.97 (−5.53 to 7.47)	3.11	2.61 (0.26 to 4.95)	1.14
Prefer to not answer	−0.42 (−6.37 to 5.53)	2.84	−0.27 (−5.00 to 4.47)	2.26
**Health insurance**				
Yes	1 [Reference]		1 [Reference]	
No	0.26 (−1.49 to 2.01)	0.83	0.27 (−1.09 to 1.63)	0.65
**Program type**				
For-profit center	1 [Reference]		1 [Reference]	
Home-based/family childcare	−2.30 (−3.89 to −0.72)^b^	0.76	−1.54 (−2.45 to −0.63)^b^	0.43
Nanny/home visiting	−0.49 (−3.70 to 2.73)	1.54	−0.20 (−3.03 to 2.63)	1.35
Nonprofit agency center	−0.02 (−1.18 to 1.13)	0.55	0.30 (−0.47 to 1.07)	0.37
School-based	−0.40 (−2.10 to 1.30)	0.81	0.11 (−0.87 to 1.09)	0.47
Head Start/Early Head Start	−0.73 (−2.77 to 1.30)	0.97	−0.07 (−1.24 to 1.10)	0.56
Drop-in center	−0.27 (−4.06 to 3.52)	1.81	0.73 (−2.15 to 3.61)	1.38
Other center-based	−0.68 (−2.58 to 1.22)	0.91	−0.13 (−1.59 to 1.33)	0.70

Abbreviations: Abbreviations: CES-D-10, Epidemiological Studies–Depression-10; PSS-10, Perceived Stress Scale-10.

a Data were collected from self-identifying childcare professionals through an online Qualtrics survey distributed from May 22, 2020, through June 8, 2020.

b *P* < .05; determined by linear regression.

#### Stress

Of the total sample, 33.4% (n = 27,317) of respondents reported low stress levels, 42.5% (n = 34,752) reported moderate stress levels, and 24.0% (n = 19,629) reported high stress levels, with a mean score of 17.5 (SD, 7.4) (Table 3). For every 1-year increase in age, on average, the PSS-10 summary score decreased (β = −0.07; 95% CI, −0.11 to −0.03) (Table 4). Compared with participants who worked in for-profit centers, participants in home-based programs reported lower PSS-10 summary scores (β = −1.54; 95% CI, −2.45 to −0.63) while controlling for other covariates (Table 4).

## Discussion

In our study, the largest national study of the physical and mental health of US childcare professionals to date, the prevalence of depression was higher among childcare professionals during the COVID-19 pandemic than before the pandemic, and asthma, stress, and depression rates were higher among childcare professionals than among US adult overall during the pandemic. Additionally, race, ethnicity, and sex/gender disparities were found for physical health conditions but not mental health conditions of childcare professionals during the pandemic. Our findings highlight a need for effective supports for the overall well-being of this socially vulnerable, yet essential, workforce.

The depression rate for childcare professionals (45.7%) 2 or 3 months into the COVID-19 pandemic was greater than estimates for childcare professionals before the pandemic (16.0% to 36.1%) (11–15), and greater than estimates for US adults during the pandemic (27.8% to 32.8%) (19–21). Approximately two-thirds (66.5%) of childcare professionals reported moderate (42.5%) or high (24.0%) stress levels, almost twice the estimate for US adults during the pandemic (22). This rate of stress among childcare professionals is greater than prepandemic rates for childcare professionals (7,10,23) and similar to rates during the early months of the pandemic reported by others (7).

The increased levels of stress and depressive symptoms may be due to the challenging working conditions that childcare professionals face, including low wages. Although these conditions existed before the COVID-19 pandemic, they have been exacerbated by the pandemic. Childcare professionals’ compensation decreased at the beginning of the pandemic because of low child enrollment (7), and low compensation has been linked to increases in burnout and stress (33). The increased levels of stress and depressive symptoms found in our study are concerning because poor mental health status among childcare professionals is associated with increased teacher–child conflicts and negative social–emotional teaching (23). Another explanation for the increased levels of stress and depressive symptoms may be the decrease in physical activity and mobility and increase in sedentary behavior, as childcare professionals adapted to the COVID-19 pandemic lockdowns, which were found to be associated with worse mental health outcomes (34–36). Overall, identifying mental health interventions that support the well-being of childcare professionals is an important step toward improving the quality of childcare programs and children’s academic and personal development.

Across the 10 medical conditions identified by the Centers for Disease Control and Prevention near the beginning of the pandemic as risk factors for COVID-19 complications (25), 26.2% of survey respondents reported 1 condition, 9.7% reported 2 conditions, and 4.0% reported 3 or more conditions. Asthma rates among childcare professionals in this study (14.3%) were about 1.2 times the national average among US women (37). Our finding on the high rate of asthma among childcare professionals requires immediate attention because people with asthma are more likely than people without asthma to be hospitalized for COVID-19 (38). Also, childcare centers are often poorly ventilated and have high levels of indoor air pollutants (39), suggesting the need to monitor air quality in childcare facilities to protect the health of childcare professionals and reduce their vulnerability to health complications. In contrast to the high rate of asthma among childcare professionals, the rates for diabetes (6.5%), heart disease (4.9%), and obesity (19.8%) among childcare professionals were below national rates for US adult women (16,17,40).

Race, ethnicity, and sex/gender disparities were evidenced for physical health conditions of childcare professionals but not for mental health during the pandemic. Compared with White childcare professionals, those who identified as either American Indian/Native Alaskan or Black/African American had increased odds for asthma, heart disease, and diabetes, and multiracial childcare professionals had increased odds of asthma, heart disease, and obesity. Both findings are consistent with the racial and ethnic disparities among US adult women (17,37,40). Illustrating these disparities, 7.4% of American Indian/Native Alaska childcare professionals reported 3 or more chronic health conditions that place them at greater risk of COVID-19 complications, compared with 4.0% among childcare professionals overall. Of the physical health conditions examined, diabetes showed the greatest level of disparities, with all racial groups (except multiracial) and the Hispanic group showing increased odds when compared with White childcare professionals, which is consistent with racial and ethnic disparities among US adult women (41). Also, childcare professionals who reported nonbinary gender identity had increased odds of both asthma and heart disease compared with female childcare professionals.

Although our study found racial and ethnic disparities in the rates of chronic diseases, the reason for these differences may not be simply racial and ethnic minority status but also socioeconomic status. With an average salary of $16,443 (as of 2019) (4), childcare professionals are economically disadvantaged. However, childcare professionals of color are paid considerably less per hour than their White counterparts, even when controlling for educational level (42). Therefore, the higher prevalence of chronic diseases, especially heart disease, diabetes, and obesity, among childcare providers of color, who are likely to come from low-income communities, may be due to poor nutritional habits, lack of adequate exercise, and other behavioral and environmental factors that are associated with low-income status (43).

Professionals working in the federally funded Head Start or Early Head Start program had greater odds for diabetes and obesity, compared with professionals working in for-profit childcare centers, even when controlling for sociodemographic characteristics. Childcare professionals with asthma, diabetes, or obesity were more likely to have access to medical insurance, regardless of age or other sociodemographic characteristics, perhaps explainable by previous findings showing that people with medical insurance are more likely to use basic clinical services and therefore more likely to receive a diagnosis and treatment from a primary care provider (44).

### Strengths and limitations

The major strength of our study is that it is a large national sample weighted to representativeness, allowing robust estimates of US childcare professionals’ physical and mental health status and enough statistical power to explore subgroup conditions. The greatest methodologic limitation is the sole reliance on self-reported information, without medical or psychiatric examination to verify the reporting. Also, findings were obtained during the early months of the COVID-19 pandemic (May–June 2020), and the mental health impacts of the COVID-19 pandemic may have changed after that time. Lastly, our data do not contribute to identifying interventions that may help manage the high levels of stress and depressive symptoms among childcare professionals.

### Conclusion

Given the impacts of the pandemic on this essential workforce, efforts should be directed toward developing effective and scalable interventions for improving the physical and mental health of childcare professionals and addressing stressors that may undermine their well-being. Our findings emphasize the need to further examine the health behaviors of childcare professionals, via mixed-methods research, to identify health initiatives that might improve their overall health.

## Appendix Supplemental Tables

**Table:** Supplemental Table 1. Prevalence of 6 Physical Health Outcomes, by Sociodemographic Characteristics, Among US Childcare Professionals, 2020^a^

Characteristic	Total	Chronic lung disease/chronic obstructive pulmonary disease	Smoking	Chronic/severe kidney disease	Liver disease	Immunosuppressive treatments of cancer/inflammatory disease	Immune-compromising conditions
**Total**	81,682	814 (1.0)	3,619 (4.4)	562 (0.7)	545 (0.7)	3,851 (4.7)	1,884 (2.3)
**Age, mean (SD), y**	42.1 (14.1)	54.0 (13.0)	39.7 (13.4)	48.6 (14.9)	46.1 (13.4)	47.4 (13.1)	40.5 (14.0)
**Race**							
American Indian/Alaska Native	1,582 (1.9)	33 (2.1)	110 (7.0)	26 (1.6)	31 (2.0)	74 (4.7)	41 (2.6)
Asian	2,949 (3.6)	12 (0.4)	43 (1.4)	10 (0.3)	12 (0.4)	67 (2.3)	17 (0.6)
Black/African American	11,837 (14.5)	108 (0.9)	484 (4.1)	82 (0.7)	55 (0.5)	559 (4.7)	192 (1.6)
Multiracial	2,944 (3.6)	18 (0.6)	186 (6.3)	13 (0.4)	22 (0.8)	138 (4.7)	108 (3.7)
Native Hawaiian/Pacific Islander	491 (0.6)	12 (2.4)	26 (5.3)	2 (0.3)	3 (0.5)	24 (4.9)	17 (3.4)
Prefer to not answer	9,731 (11.9)	57 (0.6)	260 (2.7)	56 (0.6)	75 (0.8)	371 (3.8)	141 (1.5)
White	52,164 (63.8)	575 (1.1)	2,510 (4.8)	375 (0.7)	348 (0.7)	2,617 (5.0)	1,369 (2.6)
**Hispanic**							
No	61,806 (75.7)	714 (1.2)	3,081 (5.0)	450 (0.7)	381 (0.6)	3,151 (5.1)	1,573 (2.5)
Yes	17,753 (21.7)	69 (0.4)	466 (2.6)	104 (0.6)	144 (0.8)	613 (3.5)	271 (1.5)
Prefer to not answer	2,139 (2.6)	30 (1.4)	72 (3.4)	8 (0.4)	21 (1.0)	87 (4.0)	40 (1.9)
**Sex/gender**							
Female	78,725 (96.4)	786 (1.0)	3,422 (4.3)	534 (0.7)	498 (0.6)	3,759 (4.8)	1,811 (2.3)
Male	2,033 (2.5)	12 (0.6)	135 (6.6)	19 (0.9)	29 (1.4)	41 (2.0)	38 (1.9)
Nonbinary	225 (0.3)	5 (2.1)	38 (16.7)	8 (3.4)	0 (0)	22 (9.7)	27 (12.0)
Prefer to not answer	715 (0.9)	12 (1.7)	25 (3.4)	2 (0.2)	3 (0.5)	29 (4.0)	9 (1.3)
**Health insurance**							
No	8,808 (10.8)	64 (0.7)	569 (6.5)	42 (0.5)	49 (0.6)	253 (2.9)	136 (1.5)
Yes	72,890 (89.2)	750 (1.0)	3,050 (4.2)	520 (0.7)	496 (0.7)	3,598 (4.9)	1,749 (2.4)
**Program type**							
For-profit center	19,976 (24.5)	203 (1.0)	1,112 (5.6)	146 (0.7)	119 (0.6)	946 (4.7)	521 (2.6)
Home-based/family childcare	18,078 (22.1)	214 (1.2)	500 (2.8)	127 (0.7)	120 (0.7)	922 (5.1)	339 (1.9)
Nanny/home visiting	1,115 (1.4)	23 (2.1)	49 (4.4)	9 (0.8)	5 (0.5)	56 (5.0)	48 (4.3)
Nonprofit agency center	15,875 (19.4)	165 (1.0)	665 (4.2)	98 (0.6)	110 (0.7)	806 (5.1)	395 (2.5)
School-based	10,604 (13.0)	78 (0.7)	359 (3.4)	65 (0.6)	82 (0.8)	457 (4.3)	247 (2.3)
Head Start/Early Head Start	8,506 (10.4)	82 (1.0)	532 (6.3)	71 (0.8)	60 (0.7)	382 (4.5)	189 (2.2)
Drop-in center	1,596 (2.0)	8 (0.5)	134 (8.4)	3 (0.2)	10 (0.6)	44 (2.7)	20 (1.2)
Other center-based	5,949 (7.3)	40 (0.7)	268 (4.5)	44 (0.7)	39 (0.7)	239 (4.0)	124 (2.1)

^a^ Data were collected from self-identifying childcare professionals through an online Qualtrics survey distributed from May 22, 2020, through June 8, 2020. All values are number (percentage) unless otherwise indicated.

**Table:** Supplemental Table 2. Number of Chronic Diseases or Health Conditions, by Sociodemographic Characteristics, Among US Childcare Professionals, 2020^a^

Characteristic	No. of chronic diseases or health conditions			
	0	1	2	≥3
**Total**	49,099 (60.1)	21,398 (26.2)	7,962 (9.7)	3,239 (4.0)
**Age, mean (SD), y**	41.1 (13.9)	42.7 (14.1)	44.5 (13.8)	48.1 (13.7)
**Race**				
American Indian/Alaska Native	863 (54.6)	421 (26.6)	181 (11.4)	117 (7.4)
Asian	2,221 (75.3)	538 (18.3)	138 (4.7)	51 (1.7)
Black/African American	6,718 (56.8)	3271 (27.6)	1,319 (11.1)	530 (4.5)
Multiracial	1,561 (53.0)	832 (28.3)	406 (13.8)	146 (4.9)
Native Hawaiian/Pacific Islander	305 (62.1)	113 (23.1)	48 (9.7)	25 (5.1)
Prefer to not answer	6,557 (67.4)	2,214 (22.8)	697 (7.2)	263 (2.7)
White	30,874 (59.2)	14,009 (26.9)	5,174 (9.9)	2,107 (4.0)
**Hispanic**				
No	35,969 (58.2)	16,739 (27.1)	6,410 (10.4)	2,588 (4.3)
Yes	11,738 (66.1)	4,154 (23.4)	1,392 (7.8)	468 (2.6)
Prefer to not answer	1,392 (65.1)	505 (23.6)	160 (7.5)	82 (3.8)
**Sex/gender**				
Female	47,227 (60.0)	20,662 (26.2)	7,709 (9.8)	3,127 (4.0)
Male	1,300 (64.0)	498 (24.5)	169 (8.3)	65 (3.2)
Nonbinary	88 (39.2)	80 (35.6)	33 (14.9)	23 (10.3)
Prefer to not answer	484 (67.7)	157 (21.9)	51 (7.1)	23 (3.3)
**Health insurance**				
No	5,654 (64.2)	2,212 (25.1)	711 (8.1)	231 (2.6)
Yes	43,445 (59.6)	19,186 (26.3)	7,251 (9.9)	3,008 (4.1)
**Program type**				
For-profit center	11,757 (58.9)	5,359 (26.8)	2,008 (10.1)	852 (4.3)
Home-based/family childcare	11,247 (62.2)	4,438 (24.5)	1,687 (9.3)	706 (3.9)
Nanny/home visiting	717 (64.3)	246 (22.1)	103 (9.3)	48 (4.3)
Nonprofit agency center	9,171 (57.8)	4,369 (27.5)	1,635 (10.3)	699 (4.4)
School-based	6,755 (63.7)	2,692 (25.4)	844 (8.0)	313 (3.0)
Head Start/Early Head Start	4,783 (56.2)	2,369 (27.0)	972 (11.4)	381 (4.5)
Drop-in center	1,053 (66.0)	374 (26.2)	126 (7.9)	43 (2.7)
Other center-based	3,617 (60.8)	1,549 (26.0)	586 (9.8)	197 (3.3)

^a^ Data were collected from self-identifying childcare professionals through an online Qualtrics survey distributed from May 22, 2020, through June 8, 2020. All values are number (percentage) unless otherwise indicated.

## Post-Test Information

To obtain credit, you should first read the journal article. After reading the article, you should be able to answer the following, related, multiple-choice questions. To complete the questions (with a minimum 75% passing score) and earn continuing medical education (CME) credit, please go to http://www.medscape.org/journal/pcd. Credit cannot be obtained for tests completed on paper, although you may use the worksheet below to keep a record of your answers.

You must be a registered user on http://www.medscape.org. If you are not registered on http://www.medscape.org, please click on the “Register” link on the right hand side of the website.

Only one answer is correct for each question. Once you successfully answer all post-test questions, you will be able to view and/or print your certificate. For questions regarding this activity, contact the accredited provider, CME@medscape.net. For technical assistance, contact CME@medscape.net. American Medical Association’s Physician’s Recognition Award (AMA PRA) credits are accepted in the US as evidence of participation in CME activities. For further information on this award, please go to https://www.ama-assn.org. The AMA has determined that physicians not licensed in the US who participate in this CME activity are eligible for AMA PRA Category 1 Credits™. Through agreements that the AMA has made with agencies in some countries, AMA PRA credit may be acceptable as evidence of participation in CME activities. If you are not licensed in the US, please complete the questions online, print the AMA PRA CME credit certificate, and present it to your national medical association for review.

## Post-Test Questions

### Study Title: Prevalence of Chronic Diseases, Depression, and Stress Among US Childcare Professionals During the COVID-19 Pandemic

#### CME Questions

1. Which one of the following chronic illnesses was most common among childcare professionals in the current study?
  - Diabetes
  - Heart disease
  - Asthma
  - Hypothyroidism
2. Which one of the following racial/ethnic groups experienced the highest rates of asthma and cardiac disease in the current study?
  - Black/African American and Hispanic
  - Hispanic and White
  - Black/African American and Asian
  - Black/African American and American Indian/Alaska Native
3. What was the approximate rate of positive screening for depression among childcare professionals in the current study?
  - 8%
  - 15%
  - 24%
  - 46%
4. Which one of the following variables had the strongest association with higher stress levels in the current study?
  - Older age
  - Black/African American race
  - Being a woman
  - Working in a Head Start program
